# 222 nm far-UVC light markedly reduces the level of infectious airborne virus in an occupied room

**DOI:** 10.1038/s41598-024-57441-z

**Published:** 2024-03-20

**Authors:** Manuela Buonanno, Norman J. Kleiman, David Welch, Raabia Hashmi, Igor Shuryak, David J. Brenner

**Affiliations:** 1 Center for Radiological Research, Columbia University Irving Medical Center, 630 West 168th St., New York, NY 10032 USA; 2 Department of Environmental Health Sciences, Mailman School of Public Health, Columbia University Irving Medical Center, New York, NY USA

**Keywords:** Applied microbiology, Viral transmission

## Abstract

An emerging intervention for control of airborne-mediated pandemics and epidemics is whole-room far-UVC (200–235 nm). Laboratory studies have shown that 222-nm light inactivates airborne pathogens, potentially without harm to exposed occupants. While encouraging results have been reported in benchtop studies and in room-sized bioaerosol chambers, there is a need for quantitative studies of airborne pathogen reduction in occupied rooms. We quantified far-UVC mediated reduction of aerosolized murine norovirus (MNV) in an occupied mouse-cage cleaning room within an animal-care facility. Benchtop studies suggest that MNV is a conservative surrogate for airborne viruses such as influenza and coronavirus. Using four 222-nm fixtures installed in the ceiling, and staying well within current recommended regulatory limits, far-UVC reduced airborne infectious MNV by 99.8% (95% CI: 98.2–99.9%). Similar to previous room-sized bioaerosol chamber studies on far-UVC efficacy, these results suggest that aerosolized virus susceptibility is significantly higher in room-scale tests than in bench-scale laboratory studies. That said, as opposed to controlled laboratory studies, uncertainties in this study related to airflow patterns, virus residence time, and dose to the collected virus introduce uncertainty into the inactivation estimates. This study is the first to directly demonstrate far-UVC anti-microbial efficacy against airborne pathogens in an occupied indoor location.

## Introduction

Limiting airborne disease transmission in occupied public spaces is a key public health goal. Diseases such as COVID-19, seasonal influenza and tuberculosis are largely airborne transmitted, and the recent pandemic has emphasized a clear need for improved engineering controls to limit their spread.

The use of ventilation or indoor air filtration to reduce disease transmission has been much discussed; while useful in some situations, these approaches are generally flow limited, which, together with cost and energy conservation issues, has reduced their utility^1^.

One of the most promising engineering controls for preventing airborne disease transmission is the use of UVC light to directly inactivate airborne pathogens. Air disinfection by conventional germicidal UVC (peak wavelength 254 nm) was first successfully demonstrated in the 1940s^2,3^, using systems that restricted the UVC lamp emissions to the upper unoccupied volumes of occupied rooms, in order to prevent human health hazards arising from UVC overexposure^4^.

Recent research has investigated the use of far ultraviolet C (far-UVC) sources for whole-room air disinfection in occupied locations. Far-UVC, typically defined as wavelengths in the range of 200–235 nm, effectively inactivates airborne pathogens^5–7^, including SARS-CoV-2, influenza, and other airborne pathogens^8–11^, while posing negligible damage to human and rodent skin^12–22^ and eyes in animal models^23–26^ as well as in human volunteers^27–30^. The mechanistic background relates to the very limited penetration of far-UVC light into living cells in the skin and eyes^31^. In contrast, airborne pathogens have far smaller dimensions than cells, allowing far-UVC to penetrate and inactivate them. Far-UVC therefore represents a promising technology for whole room airborne disinfection in occupied indoor locations and may be an effective intervention for pandemic or epidemic control^32,33^.

Our initial laboratory studies using a custom-built benchtop aerosol chamber have shown that 222 nm far-UVC is very effective at inactivating airborne viruses such as human coronaviruses and influenza A virus^8,9^. Subsequent pioneering studies using a room-scale bioaerosol chamber^33^ outfitted with overhead 222 nm lamps have demonstrated that far-UVC at intensities below the current recommended U.S. daily limits^34^ reduced steady-state airborne *S. aureus* load by more than 98%. At far-UVC levels below the corresponding international recommended daily limits^35^, the resultant airborne pathogen reduction was more than 92%^33^.

While far-UVC installations in occupied indoor settings to reduce transmission of infectious diseases have begun to be employed^36–39^, quantitative evidence of its germicidal effectiveness in various real-life scenarios is lacking, limiting the evaluation of the utility of this technology for disinfection in human-occupied environments.

Such quantitative demonstrations of efficacy in occupied locations are not easy. A principal challenge stems from variability in sampling and culturing from the ambient environment. Quantifying changes in viable airborne viral loads is challenging, in part, because of the need for virus-specific host cells to quantify viral infectivity, which essentially requires a priori knowledge of the virus that is being captured. Even when a particular virus is thought to be present in suitable concentration, such as within a seasonal influenza epidemic, the amount of virus present will exhibit significant temporal fluctuations on time scales of minutes, hours, days, weeks and months. Thus, attempts to quantify far-UVC induced pathogen reduction are typically limited by the lack of a sufficiently robust baseline.

Efforts to introduce a known concentration of microbes for a controlled field-testing experiment in an occupied space, even using bacteriophages which are not considered harmful to humans, have been hampered by ethical and public perception concerns.

By contrast, an occupied indoor environment with a naturally occurring high airborne viral load would represent an ideal testing ground for airborne intervention methods such as far-UVC. We have identified such a location in an occupied mouse-cage cleaning room in the Columbia University animal housing facility. This mouse-cage cleaning room normally contains high airborne level of murine norovirus, MNV, a virus that is not harmful to humans. In brief, because murine norovirus MNV is endemic among laboratory mice^40^, their used cage bedding contains large quantities of MNV, and thus when the cage bedding is disposed of inside the mouse-cage cleaning room, large amounts of MNV are released into the occupied indoor air.

Here we describe a field test on the efficacy of 222 nm far-UVC to reduce pseudo steady-state murine norovirus load in air samples collected in a mouse-cage cleaning room where the air samples were collected during daily work activities of the animal husbandry staff. The consistent contamination of this space with a high concentration of airborne virus enables testing for air disinfection without the experimental introduction of a test microbe. For this field study, we used a portable air sampler and compared the ability of collected airborne MNV to infect host cells when the far-UVC sources were on or off on alternate weeks over a period of four months. In addition, we monitored potential changes in air quality (ozone and particulates) that might potentially be associated with the far-UVC exposure.

## Results

### Benchtop studies of far-UVC sensitivity of MNV *vs.* H1N1 influenza virus and OC43 human coronavirus

**Figure 1:**
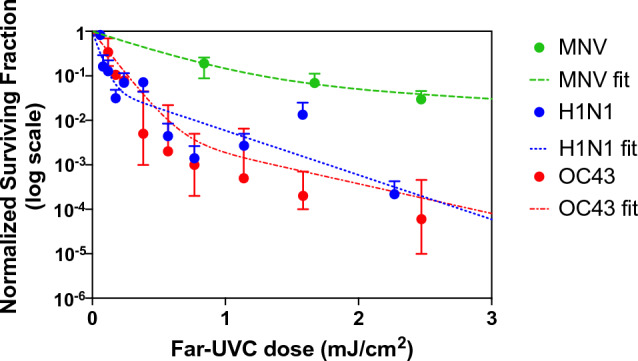
Laboratory study results on inactivation of different aerosolized viruses exposed to graded doses of 222 nm far-UVC in a benchtop aerosol chamber. Results for aerosolized MNV are compared with human influenza A H1N1virus and human OC43 coronavirus. Fractional survival TCID_50,UVC_/TCID_50, controls_ are plotted as a function of the 222-nm far-UVC dose. Values are reported as mean ± SD (n = 3) and the curves represents the best-fit robust bi-exponential regression (Eq. 1).

Our goal in these initial benchtop studies was to assess the utility of airborne MNV as a surrogate for common airborne viruses such as human influenza virus and coronavirus. In our benchtop system^8,9,41^ we generated survival curves for aerosolized MNV and aerosolized H1N1 influenza virus and compared the results to those obtained previously for aerosolized human coronavirus OC43^41^. The results are shown in Fig. 1. Analysis of the data based on Eq. (1) (Table 1) indicates that in the case of MNV the inactivation rate constant for the sensitive population was k_1,MNV_ = 2.36 ± 0.63 cm^2^ mJ^−1^, corresponding to a dose required to kill 90% of the exposed viruses D_90,MNV_ = 0.98 mJ cm^−2^. For human influenza A (H1N1), k_1,H1N1_ = 20.06 ± 8.04 cm^2^ mJ^−1^, corresponding to D_90,H1N1_ = 0.11 mJ cm^−2^. Previous studies with human beta coronavirus OC43^9^ yielded k_1,OC43_ = 12.4 ± 0.4 cm^2^ mJ^−1^, corresponding to D_90,OC43_ = 0.19 mJ cm^−2^ (Table 1).

**Table 1:** Best-fit parameters of the bi-exponential model (Eq. 1) of measured airborne MNV and human influenza A (H1N1) dose-dependent surviving fraction.

Virus (strain)	Best-fit parameter estimate ± SE			D_90_ (mJ cm^−2^)
	k_1_ susceptibility constant for “sensitive” population (cm^2^ mJ^−1^)	k_2_, susceptibility constant for “resistant” population (cm^2^ mJ^−1^)	% resistant population	
Murine norovirus (CW1)	2.36 ± 0.63	0.35 ± 0.06	8.5 ± 4.6	0.98
Human influenza A (H1N1) (A/PR/8/34)	20.06 ± 8.04	2.30 ± 0.62	6.0 ± 4.0	0.11
Human betacoronavirus 1 (OC43)	12.4 ± 0.04	1.6 ± 0.1	0.31 ± 0.054	0.19

The experiments were performed in a benchtop system with the viruses exposed to different doses of 222 nm far-UVC light. Values for human coronavirus OC43 were obtained previously^41^.

These results suggest that aerosolized MNV is comparable but somewhat less sensitive to far-UVC, as compared with aerosolized H1N1 influenza virus and human coronavirus OC43. Thus, aerosolized MNV can be considered a conservative surrogate of airborne human viruses such as influenza A and human coronaviruses^8,9^ and thus useful to investigate far-UVC airborne anti-viral efficacy.

### Inactivation of aerosolized MNV exposed to 222 nm far-UVC in an occupied mouse-cage cleaning room

We tested the efficacy of the overhead 222 nm lights in the mouse-cage cleaning room to reduce the load of MNV in the room air, using a portable air sampler with gelatin filters to collect airborne MNV particles.

**Figure 2:**
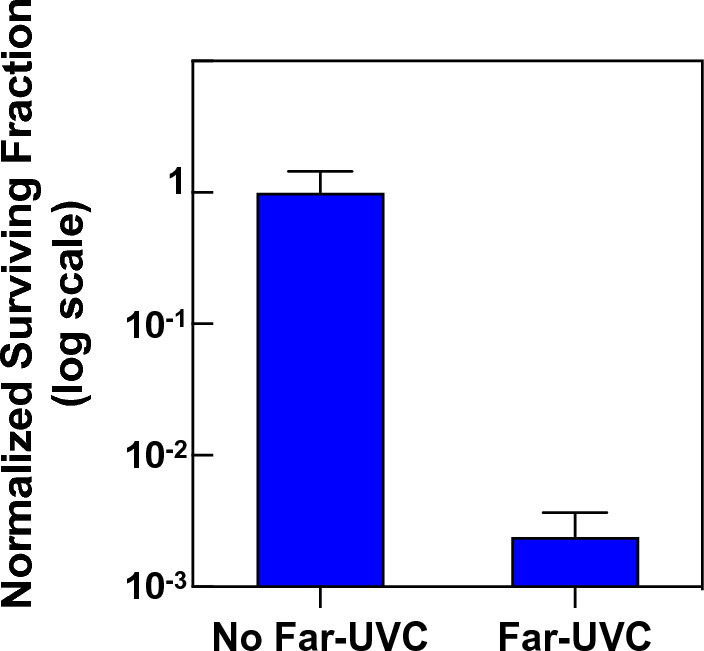
Measured reduction of pseudo steady-state murine norovirus (MNV) load in air samples collected in a mouse-cage changing room during routine work activities of the animal husbandry staff. Normalized MNV surviving fraction are shown on a log scale with far-UVC (n = 12 measurements) or without far-UVC (n = 8 measurements) ± SD.

We used the TCID_50_ assay^42,43^ to determine MNV infectivity in RAW 264.7 macrophage host cells. In the robust regression analysis of the data, all variables other than far-UVC lamps (on *vs.* off), specifically Month, Year, Time, and Lunchbreak, did not reach statistical significance and were dropped from the model. The analysis yielded a statistically significant decrease (p = 1.2 × 10^–5^) in the relative numbers of active virus for far-UVC lamps on *vs.* off corresponding to a 99.8% [95% CI: 98.2–99.9] reduction in infectious airborne MNV or a 412-fold reduction (Fig. 2). We point out that the 95% confidence interval represents the uncertainty in the measured reduction of aerosolized MNV between samples collected with the lamps on *vs.* off.

### Air quality measurements

Time-series measurements of the ambient ozone and size dependent airborne particulate matter before, during and after the far-UVC exposures are provided in the Supplemental Materials (Figs. S2 and S3). Using a Bayesian structural time series analysis, we found no evidence of causal effects of the far-UVC exposure on either the ozone measurements or any of the particulate matter measurements (p = 0.05). This data is provided with the caveat that given the high air exchange rate for the room (36 ACH based on the supply airflow and room volume) a change in air quality would not be expected to be observed.

### Room air exchange assessment

Results from the measurement of carbon dioxide decay following controlled release indicated an average air changes per hour of the tracer (ACH_T_) for the six positions measured within the room was 49 ± 20 which agrees with the reported air exchange rate of 36 for the room. Complete results are included in the Supplemental Materials Fig. S1.

The equivalent air changes per hour (eACH) provided by the far-UVC lamps given the ACH of 36, calculated using only the supply airflow and room volume, is 14,800 (95% CI: 2000–36,000). However, the layout of the supply and exhaust locations in the room suggests the room has poor mixing^44^, so the assumption of a perfectly well-mixed single compartment model is not appropriate. For example, the four supply diffusers are in the ceiling towards the center of the room and air is primarily exhausted through a double door on one side of the room, undoubtedly result in short circuiting of the flow and a formation of dead spaces. Because our sampling location is on the opposite side of the room from the door (towards which much of the supplied air will streamline) poor mixing will be especially notable in the vicinity of our sampling location. Additional details on the room airflow configuration are provided in the Supplemental Materials (Fig. S4). A common approach to account for poor mixing is to apply a mixing factor, K, which scales the ventilation for the room as in Eq. (3). When computing the eACH of a poorly mixed room a mixing factor of 5 or 10 is typically assigned^45,46^. The mixing factor decreases the effective ACH in the room from 36 to 7.2 or 3.6, and thus the estimated eACH becomes 2960 (95% C.I.: 400–7200) for a mixing factor of 5, and 1480 (95% C.I.: 200–3600) for a mixing factor of 10.

## Discussion

A number of laboratories have shown that far-UVC has a high germicidal efficacy^6,47^ within current regulatory limits, particularly for airborne pathogens, and represents minimal short- and long-term risk to skin and eyes^18,21,26^. Thus, whole room far-UVC is a promising technology to reduce transmission of infectious diseases in human-occupied spaces^32,38,48^. Its high germicidal efficacy makes far-UVC particularly promising for indoor use in the context of highly transmissible airborne viruses such as the SARS-CoV-2 Omicron subvariant^1^.

Following the suggestion—based on fundamental biophysical principles—that far-UVC might have these advantageous properties, initial efficacy studies were undertaken using a benchtop aerosol chamber^9,33,41^, and were subsequently extended to far-UVC air disinfection studies in a room-sized bioaerosol chamber with a continuous source of airborne pathogens. The room-sized bioaerosol chamber study showed that levels of far-UVC that are below the current national and international regulatory safety recommendations^34,35^ resulted in disinfection rates that were significantly higher than those provided by most other air-cleaning technologies^1^.

Although indoor installation of far-UVC sources has started in a number of human-occupied locations^36–39^, quantitative measurements of its germicidal efficacy in occupied locations are clearly necessary to evaluate the utility of this technology. This report describes the first such quantitative efficacy study in an occupied indoor location containing high levels of an airborne virus.

The airborne virus studied here is murine norovirus (MNV), which does not represent a human health hazard, but is present at high levels in the air of occupied mouse-cage cleaning rooms in animal care facilities. We first showed in a benchtop study that airborne MNV is quite similar (though slightly less sensitive) in term of its sensitivity to far-UVC (Fig. 1), as compared with airborne human influenza virus (H1N1) and airborne human coronavirus (OC43). Thus, airborne MNV represents a conservative model for assessing far-UVC germicidal efficacy for airborne human influenza virus and coronavirus.

Assuming a pseudo steady-state introduction of aerosolized MNV during routine work of the husbandry staff, we showed that four ceiling-mounted 222-nm far-UVC sources, operated at well within current regulatory limits, reduced the level of infectious airborne MNV by 99.8% [95% CI: 98.2–99.9], a 412-fold reduction (Fig. 2). These reductions are much larger than is generally achievable with ventilation and filtration^1^. However, we note that extrapolation of these results for evaluation of the susceptibility of aerosolized MNV is not straightforward since irregular airflow patterns, radiation inhomogeneity, and aerosol residence time introduce uncertainty into the dose delivered to the aerosolized virus.

Calculating the eACH using only supply ventilation airflow rates and the room volume results in the notably high value of 14,800 [95% CI: 2000–36,000] for this far-UVC lamp installation. However, the assumption of perfect mixing that is necessary for computing eACH in this manner appears inappropriate for this ventilation scenario. Instead, an estimate of eACH which incorporates a K mixing factor of 5 or 10 is applicable due to the poor mixing in the space. The assumption of poor mixing is rooted in the ventilation configuration, shown in Supplemental Fig. S4, with conditions such as the proximity of the supply to the exhaust in the ceiling and a large flow of air from the ceiling to one side of the room. This layout is undoubtedly causing short circuiting of the airflow and the formation of dead spaces, resulting in poor mixing in much of the room volume. Typically, the consideration for a ventilation layout and a K mixing factor would be applied during the design process and room configurations would be changed to avoid situations with poor mixing. Using the K mixing factor in the calculations of eACH is a simple adjustment to the ventilation rates to account for imperfect mixing within this real-world environment. The eACH due to far-UVC exposure is therefore better estimated to be 2960 (95% C.I.: 400–7200) using a mixing factor of 5, or 1480 (95% C.I.: 200–3600) using a mixing factor of 10.

Reviewing these eACH values in the context of other studies which evaluated germicidal UV systems for air disinfection is important. For instance, our previous study in a room-scale chamber examining far-UVC efficacy against aerosolized *S. aureus* resulted in an eACH of 184 [95% CI: 128–322]^33^. Similarly, a study by McDevitt et al.^49^ reported eACH values ranging up to 1000 [95% CI: 740–1400] using an upper-room UVC system. Applying a K mixing factor of 10 to the results of this study yields an eACH estimate of 1480 which is more closely aligned to these previous studies of UVC systems, especially when noting that the 95% confidence interval for this value extends down to 200.

Important in the consideration of the results of this study is the disconnect between room scale testing results and results in a benchtop aerosol chamber. Using the chamber derived k_1_ value of 2.36 cm^2^ mJ^−1^ in this study would indicate an average irradiance within the room of 1700 µW cm^−2^ [95% CI: 230–4200 µW cm^−2^]. When utilizing a K mixing value of 10 and calculating the average irradiance the value drops to 170 µW cm^−2^ [95% CI: 23–420 µW cm^−2^]. These values are, of course, much higher than any of the measured values of irradiance performed in this work. A similar discrepancy was present in the previous room-scale study with far-UVC^33^ with the computed eACH of 184 requiring an average irradiance of about 42 µW cm^−2^, which is much larger than the measured or modelled irradiance within that space. One possible cause for inconsistencies in these results is an underestimation of the k_1_ values in the benchtop chamber studies. Both the Eadie et al.^33^ paper and this work compared real-world measurements with aerosol chamber inactivation studies performed using the same chamber. The chamber has been simulated previously using computational fluid dynamic models^41^ and operates with laminar flow conditions, whereas studies in the room scale chambers are presumably more turbulent. A more complex, tumbling flow by the aerosol through an exposure area could result in higher susceptibility for the virus in the cage-cleaning room^50^. Furthermore, the exact airflow pattern for this space is uncertain, so irregularities could exist which cause variations in total dose to an aerosolized virus. The method of aerosol generation in the benchtop chamber experiments is also different than in the room scale study. The exact means of aerosolization of MNV in the cage-wash room is unknown, however the benchtop studies used a nebulizer producing droplets of similar size and composition, and variations from these conditions could alter the virus susceptibility. The discrepancy between results in a small chamber and a larger chamber/room requires further investigation.

An additional important consideration of these inactivation results is the influence of different mechanisms of inactivation for 222 nm compared to traditional UV 254 nm exposure. Direct UV damage to the viral genome is thought to be the major mechanism for the observed viral inactivation with 254 nm exposure. However, there is much evidence in the literature, dating back to the 1960s^51^, that, at far-UVC wavelengths, the dominant mode of inactivation is not direct absorbance of UV photons in the viral genome. An example is the wavelength-dependent study by Beck et al.^52^, in which they measured infectivity of an RNA virus as a function of UVC wavelength and showed that around 220 nm viral infectivity sensitivity did not track with RNA absorbance of the UV photons, but was in fact far larger. The mechanism that Beck et al., proposed (and which had also been proposed by Yarus and Sinsheimer^51^) was of the far-UVC photon energy being preferentially absorbed by proteins in the virus, followed by direct energy transfer to the RNA, resulting in site-specific RNA damage and increased loss of viral infectivity. A related mechanism that was proposed is that the UV-induced protein damage can extend to spatially-proximal RNA, causing covalent RNA–protein cross links or single-stranded breaks. More recently there has been increasing interest in experimentally characterizing the RNA-binding proteome^53^, which would allow these mechanistic suggestions to be further explored. Similar results have been reported for DNA viruses^51,54^, again showing that at far-UVC wavelengths, loss of viral infectivity is far larger than would be predicted based on direct far-UVC energy absorption in the genome, and again suggesting that the primary inactivation mechanism originates with far-UVC energy absorption in the viral protein. Supporting this suggestion, there is also experimental evidence from DNA virus studies that protein-associated DNA is much more sensitive to UVC damage as compared with isolated DNA^55,56^. Further investigations into the mechanisms of far-UVC inactivation of viruses would help in making comparisons between viral inactivation using different wavelengths of UVC.

This study also includes air-quality measurements of airborne particle concentrations and ozone concentrations, making comparisons with and without the far-UVC lamps operating. The results (Figs. S2 and S3 and Table S1) do not suggest any difference in these air quality metrics associated with far-UVC use within this space—though further air quality studies in less well-ventilated rooms are warranted.

In summary, we have shown for the first time that far-UVC light, operating well within current regulatory limits, is able to produce major reductions in the level of airborne viruses in an occupied indoor location. However, unlike controlled laboratory studies, uncertainties related to airflow pattern, residence time, and actual exposure dose experienced by the collected virus introduces uncertainty into the inactivation estimates.

Whole-room disinfection with far-UVC is an emerging intervention for pandemic and epidemic control that has the potential to become part of the layered strategic approach^57^ to minimize transmission of airborne pathogens in occupied indoor spaces, including ventilation and filtration and, where appropriate, masks and physical distancing.

## Methods

### Murine Norovirus (MNV)

Noroviruses are non-enveloped, single-stranded, positive-sense RNA viruses in the *Caliciviridae* family and have been reported in humans and many animals^58^, including laboratory mice^59^. MNV is not associated with human disease, but periodic health surveillance of laboratory animals and several large-scale studies have confirmed a high prevalence of MNV infection in laboratory mice worldwide^40,58,60^.

Airborne transmission of viruses occurs through primary aerosolization when infected cells shed viruses directly into the surrounding air or into fluids and surfaces; surfaces in turn can become sources of airborne transmission through secondary aerosolization when air movements around contaminated surfaces (fomites) or fluids disperse the viruses back into the air^61^. While transmission of MNV among laboratory mice occurs mostly by the fecal–oral route, fomites such as cages and bedding typically become colonized with MNV. At the Columbia University animal facilities, cage bedding is typically changed within dedicated mouse-cage cleaning rooms (a ~ 3500 cages are sanitized daily at Columbia). This task involves manually discarding soiled bedding from used mouse cages, in the course of which MNV in the bedding becomes aerosolized and circulated into the room air.

### Far-UVC virus susceptibility tests in benchtop aerosol chamber

In order to assess the utility of airborne MNV as a surrogate for airborne pathogens such as influenza and coronaviruses, we used our custom-built benchtop aerosol irradiation chamber to assess far-UVC sensitivity of airborne MNV and compared this with corresponding measurements with airborne influenza virus and airborne human coronavirus.

The layout and operation of this benchtop aerosol exposure system was previously described in detail^8,9,41^. This one-pass exposure system integrates the generation, exposure, and collection of virus-containing aerosols within a single chamber. The benchtop system includes a nebulizer for aerosol generation, dry and humidified air inputs to maintain humidity, particle size monitoring, an exposure volume (279 mm tall × 254 mm wide × 63 mm deep) with a UV transmitting window to enable UV exposure within the chamber, and a vacuum pump to move the aerosol through the system. Aerosol collection was performed using 37 mm gelatin membrane filters (SKC Inc., Eighty Four, PA, USA) held within a plastic air monitoring cassette (37 mm SureSeal Casette, SKC Inc.). A precision flow orifice (B-47-SS, O’Keefe Controls Co, Monroe, CT, USA) was placed immediately prior to the system vacuum pump to set the flow rate through the system to 11.6 LPM via choked flow operation conditions. The time for a particle to traverse the UV exposure window was approximately 23 s.

As in our previous studies, the average temperature during testing was 24 °C, the relative humidity was between 60% and 70%, and the aerosol size distribution was typical of human coughing, breathing, and talking^62^, with over 90% of particles less than 1.0 µm in diameter.

The far-UVC lamp used for the laboratory tests was a 12 W 222-nm KrCl excimer lamp module made by USHIO America (Item #9101711, Cypress, CA). The lamp is equipped with a proprietary optical filtering window to reduce lamp emissions outside of the 222 nm KrCl emission peak^14^. The exposure setup, lamp characteristics, and dosimetry were previously described in detail^41^. As with the previous tests using this experimental setup, precision mesh screens were used to uniformly decrease the irradiance across the exposure area to allow testing with different exposure doses.

In this study we used the murine norovirus strain MNV-1 (ATCC VR-1937) and influenza A (H1N1, A/PR/8/34, ATCC VR-95); their infectivity as function of 222 nm dose was tested, respectively, using RAW 264.7 macrophage host cells (ATCC TIB-71) and Madin-Darby canine kidney (MDCK, ATCC CCL-3) host cells, with methods previously described^9^.

Each experiment used a viral solution consisting of 1 ml minimal essential medium (MEM) (Life Technologies, Grand Island, NY) containing 10^7^–10^8^ TCID_50_ of virus, 20 ml of deionized water, and 0.05 ml of Hank’s Balanced Salt Solution with calcium and magnesium (HBSS^++^)*.* Each sampling time lasted 30 min. After the sampling period was completed, the 37 mm gelatin filter (SKC Inc., Eighty Four, PA) which captured the aerosolized virus was dissolved in 5 ml of prewarmed (~ 32 °C) PBS on an orbital shaker for 5 min. This solution was then used for the virus infectivity assays.

Virus infectivity dose–response from the benchtop laboratory studies were analyzed with a bi-exponential model^50^, where one exponential describes the behavior of a susceptible fraction of the population, and the second exponential describing the response of a more resistant subpopulation:

1 $$S = (1 - f)e^{{ - k_{1} D}} + fe^{{ - k_{2} D}}$$

Where *S* is the non-inactivated (surviving) fraction of the virus and *D* is the radiant exposure dose in mJ cm^−2^. (1–*f*) and *f* are respectively the proportions of the sensitive and the resistant subpopulations whose exponential dose responses are respectively defined by parameters *k*_*1*_ and *k*_*2*_ (units of cm^2^ mJ^−1^). D_90_ values, or the dose required to inactivate 90% (1−log reduction) of the virus, were calculated using a single exponential which only uses *k*_*1*_ and assumes that microbial inactivation in a real-world scenario occurs as repeated low-intensity exposures rendering the *k*_*2*_ susceptibility constant not relevant^50^.

### Mouse-cage cleaning room and far-UVC dosimetry

**Figure 3:**
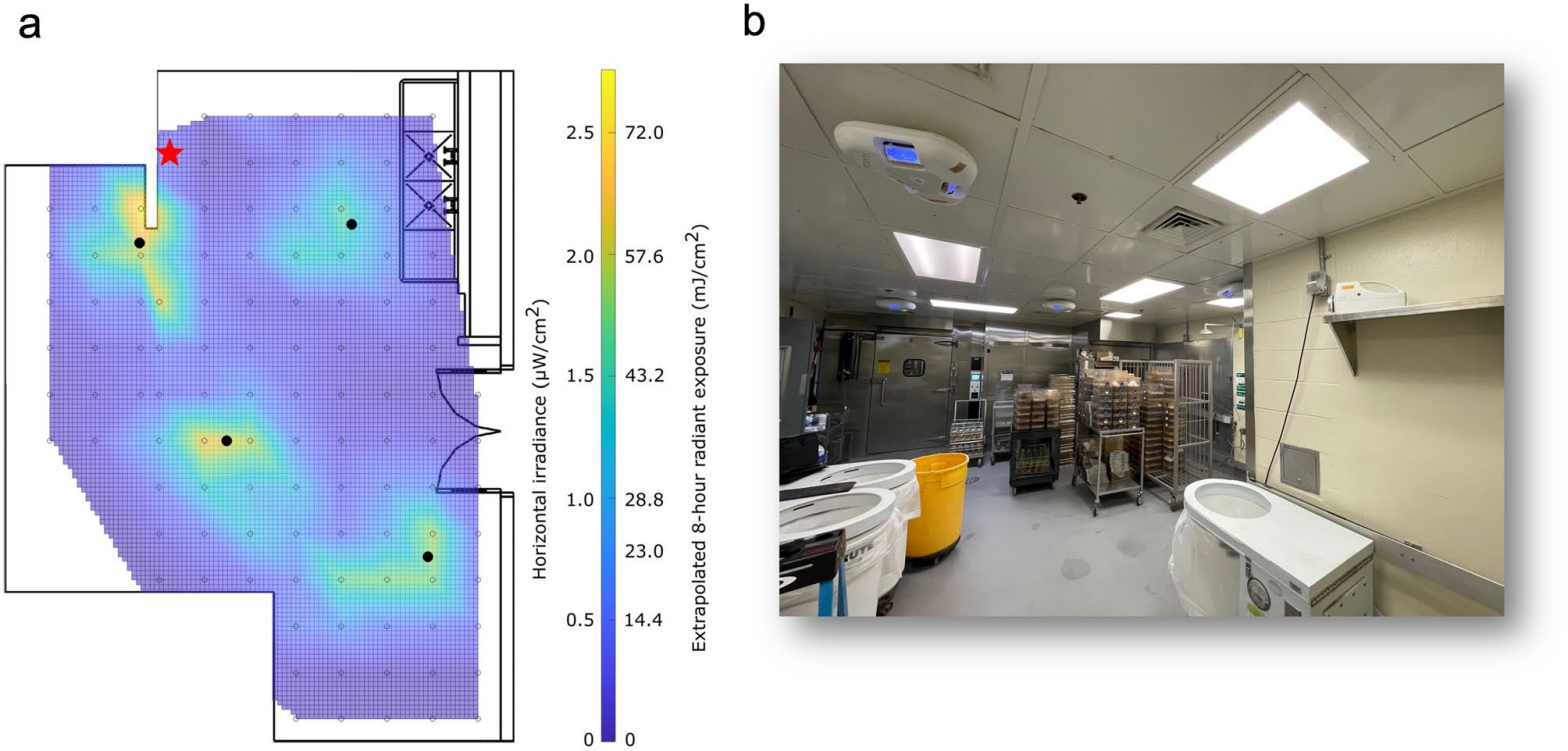
The mouse-cage changing room. (**a**) Floorplan of the mouse-cage changing room with the measured interpolated horizontal irradiance data overlaid. Horizontal irradiance was measured at 1.8 m. The locations of the four lamps on the ceiling are indicated with filled black circles. The position of the air sampler (used at 2.1 m) is marked with a star. (**b**) Photo of a section of the room showing the four far-UVC lamps turned on and the location of the shelf where the MD8 portable air sampler was located during air sampling.

222 nm far-UVC emitting lamps were installed within a mouse-cage cleaning room at the Institute of Comparative Medicine at Columbia University Irving Medical Center in New York, NY. A floorplan of the room is shown in Fig. 3a. A layout of the room showing air supply and exhaust locations is provided in the Supplemental Materials (Fig. S4). The room has a floor area of approximately 38 m^2^, a 2.5 m ceiling height, and one double door entryway which was open throughout testing. The average room temperature was 21 ± 0.8 °C and the average relative humidity was 41 ± 7.2%. Four 222 nm emitting fixtures (Vive, R-Zero Systems, South Salt Lake, Utah) were installed on the ceiling tiles of the room at the positions indicated in Fig. 3a and in the illustrative photo in Fig. 3b, which includes the four far-UVC lamps and the shelf where the portable air filter was positioned for each air sampling. Each fixture contains three KrCl bulbs and all bulbs were oriented to emit at a 45° angle outward. An optical filter is included with each bulb to reduce lamp emissions outside of the 222 nm KrCl emission peak^41^. A 5-µm thick polytetrafluoroethylene (PTFE) film (FP301050, Goodfellow Corporation, Pittsburgh, PA, USA) was placed across each bulb to spatially diffuse the output.

Horizontal irradiance was recorded at a height of 1.8 m from the floor with a UIT2400 Handheld Light Meter (Ushio America, Cypress, CA). Irradiance measurements were made at regular intervals throughout the room in a grid pattern with spacing of 0.5 m. Irradiance values and their respective positions were input into MATLAB (Mathworks, Natick, MA, USA) and the *griddata* function was used to interpolate between the points. The interpolated irradiance values for the space are plotted in Fig. 3a and are shown overlaid onto the floorplan for the room. The peak irradiance value measured at a height of 1.8 m was 2.76 µW cm^−2^ and the average irradiance was 0.464 µW cm^−2^ corresponding to an average dose over a 40 min sample collection time of 1.1 mJ cm^−2^. Extrapolating the peak irradiance value to an 8-h exposure results in a radiant exposure of 79.5 mJ cm^−2^, which is well below the American Conference of Governmental and Industrial Hygienists (ACGIH) recommended threshold limit values (TLVs) at 222 nm of 160 mJ cm^−2^ for eye exposure and 480 mJ cm^−2^ for skin exposure^34^.

A radiation model of the mouse-cage cleaning room with the far-UVC fixtures was created using Visual Lighting software (Acuity Brands, Atlanta, GA). The model was used to estimate the average fluence rate throughout the room volume as well as the average and maximum horizontal irradiance at 1.8 m up from the floor. The far-UVC fixtures were modelled using IES files supplied by the manufacturer. Reflectivity values for flooring, paint, and ceiling tile for the 222 nm radiation were specified as 7%, 8.1%, and 7.5%, respectively. Using these parameters, the model computed an average fluence for the room volume of 0.82 µW cm^−2^. The average horizontal irradiance at 1.8 m was 0.6 µW cm^−2^ and the maximum irradiance at 1.8 m was 4.8 µW cm^−2^. Extrapolating the modelled peak irradiance value to an 8-h exposure results in a radiant exposure of 140 mJ cm^−2^.

### Air sampling and virus infectivity

A total of 20 air samples were collected over a four month period, with samples collected either on Mondays or Wednesdays between the hours of 9:30 am to 1 pm. The far-UVC lamps were either on or off for alternate weeks during this time. On each air sampling day, at least four separate air samples were collected. On the sampling days with the sources turned on, the lamps were turned on approximately two hours before air collection began. Husbandry staff were present and active for all sampling days.

To sample active MNV airborne concentrations, a portable air sampler (MD8, Sartorius,) was equipped with a water-soluble 80-mm disposable gelatin filter (Sartorius, 17528-80ACD), which protects the viruses from drying during collection, thus ensuring high retention. The air sampler was set to operate at 50 LPM (liter per minute) and each air sample collection lasted 40 min such that the total air volume filtered per sample was 2000 L. During sampling, the unit was placed on a shelf at a height of 2.1 m at the position in the room indicated by the star in Fig. 3a and visible in the photo of the room in Fig. 3b.

After each air sampling period, the gelatin filter was placed in 6 ml of prewarmed (~ 37 °C) MNV infectious medium, allowed to dissolve in the incubator for 15 min, and then maintained at 37 °C. The viral solutions were then transported in a portable incubator set at 37 °C to our BSL 2 facility located at a ~ 30 min drive from the animal facility. Each sample was aliquoted for the virus infectivity assay.

### Airborne virus infectivity assay

For both the laboratory and filed-test studies, we used the 50% tissue culture infectious dose assay (TCID_50_) to determine viral infectivity^43^. Briefly, ~ 10^4^ host cells were plated in each well of 96-well plates the day prior the experiment. Cells were washed once in PBS and at least six serial 1:10 dilutions (8 wells for each dilution) in infection medium of the exposed virus from the dissolved gelatin filter was overlaid on host cells for two hours. The cells were then washed once in PBS, covered with fresh infection medium, and incubated for three or four days at 34 °C. Cytopathic effects (CPE) were scored using a bright field microscope (10×) as vacuolization of cytoplasm, cell rounding and sloughing, and the TCID_50_ was calculated using standard methodology^43^.

For data analysis, the variables far-UVC exposure (on or off), Month, Year, Lunchbreak and Time of Day were each considered as potential predictors of log-transformed virus counts. Those predictor variables that were not found to contribute significantly to the model were sequentially discarded based on the Robust Wald Test with p-value threshold of > 0.05. Potential multiplicative interactions between retained predictors were also evaluated.

### Air quality monitoring

Our goal was to quantify potential changes in air quality that might be associated with the far-UVC exposure, specifically ozone and airborne particulates. Ambient ozone concentration was measured using a Model 202 ozone monitor (2Btechnologies, Broomfield, CO, USA), and size-dependent ambient particle concentrations were measured using a PurpleAir Flex (PurpleAir, Inc., Draper, UT, USA). Both sensors were positioned on the same shelf that was used to hold the air sampler. We note that the suitability of the PurpleAir sensor is questionable for concentrations below 1–2 µg m^−3^^63,64^ and for the detection of small particles (< 0.3 µm)^65^, so results from this device are limited in their suitability for drawing conclusions on overall particulate concentrations within the space. Similarly, given the high air exchange rate for the room a measurable change in ozone concentration would not be expected.

Ozone measurements were recorded every 10 s, and particle concentrations were recorded every two minutes. Sensors recorded data both with and without the far-UVC sources operating. Sampling was performed over a period of five hours. The lamps were off for the first two hours, then on for two hours, and finally off for the final hour of measurements. The native operation of the heating, ventilation, and air conditioning (HVAC) system was maintained during all testing. Measurements were taken during normal working hours with animal-care staff members typically present.

The air quality time-series data were analyzed to assess the possible causal impact of far-UVC exposure using a Bayesian structural time-series model^66^. We used the *CausalImpact R* package to assess potential causal effects of far UVC on the measured air-quality parameters.

The ventilation rate of the room was measured as part of the most recent Association for Assessment and Accreditation of Laboratory Animal Care International (AAALAC) facility verification procedure on December 1, 2021. The report documents a total room volume of 3,405 ft^3^ and a total measured supply airflow of 2068 CFM, for an ACH of approximately 36 if perfect mixing throughout the room volume is assumed.

### Room air exchange assessment

The mechanical ventilation for the room was characterized using controlled release experiments and the measurement of decay rate^67–69^. Analysis was performed using carbon dioxide as a tracer gas. Carbon dioxide gas was briefly released into the unoccupied room to increase the concentration throughout the space and then cleared by mechanical ventilation. The carbon dioxide concentration was measured at six locations within the room, with one position being that of the air sampler, using Aranet4 sensors (SAF Tehnika, Riga, Latvia) recording at 1-min intervals. The locations of the sensors are provided in the Supplemental Materials Fig. S1. As with previous studies using this technique^68^, the air changes per hour of the tracer (ACH_T_) was calculated as the inverse slope of the linear regression of the natural logarithm of the carbon dioxide concentration versus time. The decay experiment was repeated 10 times.

The equivalent air change rate due to the Far-UVC was calculated using

2 $$\frac{C}{{C_{UV} }} = \frac{{N + N_{UV} }}{N}$$

where *C* is the average amount of virus for samples with the lamps off and *Cuv* is the average amount of active virus for samples with the far-UVC lamps on. Here *N* is the ventilation rate of the room (using the value of 36 ACH based on the supply airflow and room volume) and *Nuv* is the equivalent air change rate (eACH) due to the Far-UVC. The derivation of Eq. (2) is provided by McDevitt et al.^49^ and assumes a well-mixed room in steady state conditions.

Since the well-mixed room condition is rarely met in a real-life ventilation scenario, one method of accounting for incomplete mixing within a space is to incorporate a mixing factor, commonly denoted as K, which can be thought of as an indication of ventilation efficacy^44^. The factor K is related to the actual ventilation rate, N, and the effective ventilation rate, N’, with the equation

3 $$N^{\prime} = \frac{N}{K}$$

Values for the K mixing factor ranges from 1, representing a situation with perfect mixing, to 10, for a poorly mixed room.

### Supplementary Information

Supplementary Information.

## Data Availability

All data generated or analyzed during this study are included in this published article.
