# Supplementary material for: 222 nm far-UVC light markedly reduces the level of infectious airborne virus in an occupied room
Source: Sci Rep. 2024 Mar 20;14:6722. doi: 10.1038/s41598-024-57441-z (PMC10954628; doi:10.1038/s41598-024-57441-z)
Supplement: Supplementary file 1 — Supplementary Information. [file 41598_2024_57441_MOESM1_ESM.docx]

**222 nm far-UVC light markedly reduces the level of infectious airborne virus in an occupied room.**

Manuela Buonanno^1a^*, Norman J. Kleiman^2a^, David Welch^1a^, Raabia Hashmi^1^, Igor Shuryak^1^ & David J. Brenner^1^

^1^ Center for Radiological Research, Columbia University Irving Medical Center, New York, NY

^2^ Department of Environmental Health Sciences, Mailman School of Public Health, Columbia University Irving Medical Center, New York, NY

| ACH_T_ using CO_2_ decay | |
| --- | --- |
| Sensor position: | ACH_T_ ± SD |
| A (air sampling position) | 72.4 ± 25.3 |
| B | 47.9 ± 11.9 |
| C | 31.5 ± 3.1 |
| D | 48.9 ± 8.9 |
| E | 59.7 ± 15.4 |
| F | 31.9 ± 8.0 |

^a^ Co-first authors equally contributed to this work.


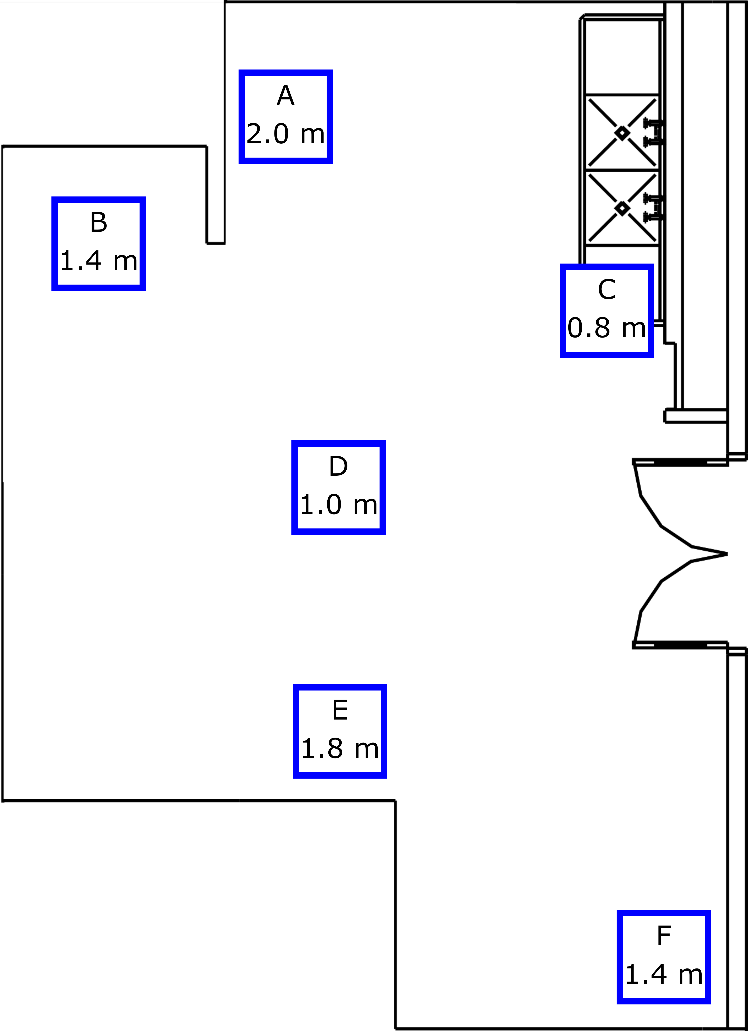


**Supplemental Fig. S1| CO_2_ decay measurement details.** The approximate positions of the CO_2_ sensors used for ACH_T_ calculations are indicated on the floorplan of the cage-cleaning room. The 6 positions are labeled A through F, and the height of each sensor above the floor is included with the label. Calculated ACH_T_ values are provided in the table (n=10). Position A is on the same shelf that was used to hold the air sampler.

**Fig. S2| Ambient ozone concentration monitored during a five-hour measurement period.** The UV lamp status during the measurement periods is noted at the top of the plot.

**Supplemental Fig. S3| Airborne particle counts acquired over a five-hour period.** The UV lamp status during the measurement periods is noted at the top of the plot. The size-specific particle count data are plotted as counts per liter of air using 6 particle-size bins ranging from >0.3 µm to >10.0 µm. The plotted data are summarized in Table S1.


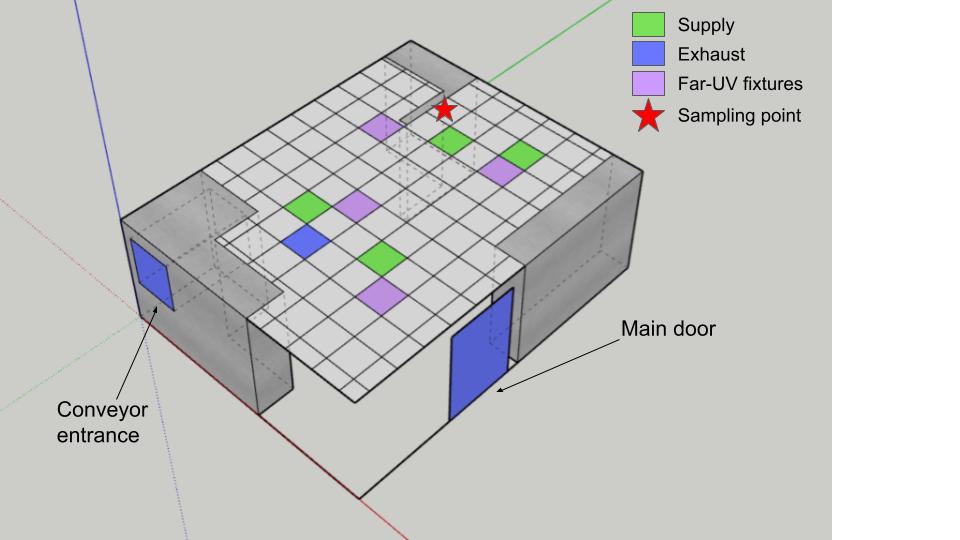


**Supplemental Fig. S4| Ventilation layout of the cage-cleaning room.** Air is supplied to the room through 4 diffusers located in the ceiling. Flow was approximately 500 CFM through each of these outlets for a total supply of 2068 CFM. Exhaust from the room was measured through a single exhaust located in the ceiling with a flow of 456 CFM or through equipment (a conveyor belt cleaning system) of 352 CFM, with the remainder of the exhaust airflow of almost 1300 CFM exiting through the double doors for the room which are always open to the hallway.

**Table S1|** **Time-averaged ozone concentration and airborne particulate matter concentrations in the mouse-cage changing room.** Data are grouped as the initial measurement period with far-UVC lamps off (2h), the subsequent measurement period with far-UVC lamps operating (2h), and the subsequent final measurement period with the far-UVC lamps off (1h). Average values for the sampling times are listed with ± standard deviation.

|  | **Ozone (ppb)** | **Particle counts (counts/L)** | | | | | |
| --- | --- | --- | --- | --- | --- | --- | --- |
|  |  | **> 0.3 µm** | **> 0.5 µm** | **> 1.0 µm** | **> 2.5 µm** | **> 5.0 µm** | **> 10.0 µm** |
| Before (no UV, 2 hr) | 25 ± 2.5 | 8200 ± 630 | 2400 ± 200 | 200 ± 35 | 10 ± 9.9 | 3.5 ± 3.7 | 1.0 ± 1.8 |
| UV on (2 hr) | 27 ± 1.6 | 8800 ± 1100 | 2600 ± 310 | 210 ± 68 | 17 ± 12 | 6.3 ± 7.3 | 2.7 ± 3.7 |
| After (no UV, 1 hr) | 25 ± 1.5 | 7500 ± 800 | 2200 ± 220 | 160 ± 45 | 8.0 ± 7.2 | 4.4 ± 5.1 | 2.5 ± 2.9 |
